# Gene-Gene Interactions in Renin-Angiotensin-Aldosterone System Contributes to End-Stage Renal Disease Susceptibility in a Han Chinese Population

**DOI:** 10.1155/2014/169798

**Published:** 2014-04-13

**Authors:** Sui-Lung Su, Hsin-Yi Yang, Chia-Chao Wu, Herng-Sheng Lee, Yuh-Feng Lin, Chi-An Hsu, Ching-Huang Lai, Chin Lin, Sen-Yeong Kao, Kuo-Cheng Lu

**Affiliations:** ^1^School of Public Health, National Defense Medical Center, Taipei, Taiwan; ^2^Division of Nephrology, Department of Medicine, Tri-Service General Hospital, National Defense Medical Center, Taipei, Taiwan; ^3^Department of Pathology, Tri-Service General Hospital, National Defense Medical Center, Taipei, Taiwan; ^4^Division of Nephrology, Department of Medicine, Shuang Ho Hospital, Graduate Institute of Clinical Medicine, Taipei Medical University, New Taipei City, Taiwan; ^5^Graduate Institute of Life Sciences, National Defense Medical Center, Taipei, Taiwan; ^6^Division of Nephrology, Department of Medicine, Cardinal Tien Hospital, School of Medicine, Fu Jen Catholic University, New Taipei City, Taiwan

## Abstract

*Objective*. In this study, we investigated whether RAAS gene single nucleotide polymorphisms (SNPs) and their interactions were associated with end-stage renal stage (ESRD). *Methodology and Results*. This was a case-control study for 647 ESRD cases and 644 controls. AGT (M235T (rs699) and T174M (rs4762)), AGTR1 (A1166C (rs5186) and C573T (rs5182)), ACE (I/D (rs1799752) and G2350A (rs4343)), and CYP11B2 C-344T (rs1799998) were genotyped and compared between cases and controls to identify SNPs associated with ESRD susceptibility. Multifactor dimensionality reduction (MDR) was used to identify gene-gene interactions. Several RAAS genes were associated with ESRD: AGT M235T, ACE I/D, ACE G2350A, and CYP11B2 C-344T. By MDR analysis, a three-locus model (ACE ID/ACE G2350A/CYP11B2 C-344T) of gene-gene interaction was the best for predicting ESRD risk, and its maximum testing accuracy was 56.08% and maximum cross-validation consistency was 9/10. ESRD risk was higher with the simultaneous occurrence of ACE I/D DD-ACE G2350A AA. AGT, ACE, and CYP11B2 gene polymorphisms are associated with ESRD. *Conclusions*. The gene-gene interaction effects of ACE I/D, ACE G2350A, and CYP11B2 C-344T polymorphisms are more important than individual factors for ESRD development among Han Chinese.

## 1. Introduction

Taiwan has the third highest incidence and the highest prevalence of end-stage renal disease (ESRD) in the world. This not only burdens healthcare resources but also has a major impact on patients and their families [1]. ESRD is a complex phenotype, which results from the presence of underlying kidney disease, and superimposing inherited and environmental factors [2]. Among the predisposing genetic factors, renin-angiotensin-aldosterone system (RAAS) disruption is clearly involved in ESRD development [3].

The RAAS is a key blood pressure, renal hemodynamics, and volume homeostasis regulator [4]. Thus, genes that encode RAAS components are candidates for evaluating predisposition to renal disease development and progression [5]. Among RAAS candidate genes, angiotensinogen (AGT), angiotensin II type I receptor (AGTR1), angiotensin-converting enzyme (ACE), and aldosterone synthase (CYP11B2) genes appear to be particularly relevant to renal disease [6].

Several studies recently identified RAAS gene mutations and polymorphisms that affected host susceptibility to several diseases, including hypertension [7], type 2 diabetes [8], myocardial infarction [9], chronic kidney disease [6, 10], and ESRD [11]. Some studies also indicated that RAAS gene single nucleotide polymorphisms (SNPs) could alter homeostasis to an abnormal state [12, 13]. RAAS gene polymorphism involvement in kidney disease pathogenesis has been extensively studied in various populations [10, 11, 14, 15]. However, the influence of interactions among RAAS genes on ESRD susceptibility remains unknown. Some RAAS genes may have strong effects as “susceptibility loci” for renal disease development and progression, whereas some may have modest effects as “modifier genes” for endophenotypic expression. Compared with a single candidate gene approach, analysis of multiple candidate gene expression variations and their interactions may be a more powerful approach for studying complex diseases.

In this study, we genotyped seven different loci in four RAAS genes in ESRD patients and healthy controls and examined gene-gene interactions using multifactor dimensionality reduction (MDR) and a logistic regression model (LRM).

## 2. Methodology

### 2.1. Subject Recruitment

This case-control study included 647 ESRD patients (346 women; 301 men; mean age, 64.4 ± 14.7 years) receiving hemodialysis at Cardinal Tien Hospital and five other hemodialysis centers in Taipei, Taiwan. These patients were stable (without clinical complications) and had undergone hemodialysis for >6 months. Autoimmune disease, malignancy, and any acute or chronic infection were exclusion criteria. ESRD causes included diabetes mellitus (*n* = 256; 39.6%), chronic glomerulonephritis (*n* = 174; 26.9%), hypertensive nephropathy (*n* = 84; 13.0%), systemic nephropathy (*n* = 66; 10.2%), and unknown (*n* = 67; 10.3%).

We also enrolled 644 healthy controls (378 women; 266 men; mean age, 65.7 ± 13.6 years) with an estimated glomerular filtration rate (eGFR) of ≥60 mL/min/1.73 m^2^ and no proteinuria recruited from the Center of Physical Examination at Cardinal Tien Hospital. They had no evidence of kidney damage, including microalbuminuria or proteinuria, hematuria, and abnormal abdominal ultrasound. Clinical information and biochemistry test results were retrieved from hospital records.

### 2.2. Ethics Statement

This study was approved by the ethics committee of Cardinal Tien Hospital (CTH-100-3-5-025). After thoroughly explaining this study, written informed consent was obtained from all participants.

### 2.3. SNP Selection and Genotyping

AGT (M235T (rs699) and T174 M (rs4762)), AGTR1 (A1166C (rs5186) and C573T (rs5182)], ACE [I/D (rs1799752) and G2350A (rs4343)), and CYP11B2 C-344T (rs1799998) polymorphisms previously shown to be significantly associated with kidney diseases in genetic polymorphism studies of Chinese Han populations [10, 16] were selected. Genomic DNA was extracted from peripheral blood samples using standard procedures with proteinase K (Invitrogen, Carlsbad, CA, USA) digestion and phenol-chloroform extraction [17]. The above mentioned polymorphisms were screened by polymerase chain reaction (PCR)-restriction fragment length polymorphism (RFLP). Primer design was based on published sequences [10, 16] or designed using primer Z software (http://genepipe.ngc.sinica.edu.tw/primerz/beginDesigndo).

PCR amplification was performed as follows. Cycling conditions were an initial denaturation at 95°C for 5 min, followed by 35 denaturation cycles at 95°C for 30 s, annealing at 55°C for 30 s, extension at 72°C for 30 s, and a final extension at 72°C for 10 min. PCR products were digested with the respective restriction endonucleases (New England Biolabs, MA, USA), and the resulting fragments were separated in 3.0% agarose gel containing 0.5 g/mL of ethidium bromide by electrophoresis at 100 V and visualized under UV light. Genotyping was performed after blinding for case or control status. Two independent investigators interpreted the images for each gel, and all ambiguous samples were analyzed again. To validate genotyping results, at least 10% of samples were randomly selected for repeated genotyping.

### 2.4. Statistical Analysis

The demographics were evaluated by Student's *t*-test for continuous variables and expressed as mean ± standard deviation (SD). Hardy-Weinberg equilibrium was assessed by a goodness-of-fit *χ* ^2^ test and performed to examine possible genotyping errors for each SNP among controls. Genotype and allele frequencies were compared between ESRD patients and healthy controls using a *χ* ^2^ test or Fisher's exact test as appropriate. LRM was used to estimate crude and adjusted (age, gender, body mass index, and smoking status) odds ratios (ORs) and 95% confidence intervals (CIs) as a measure of association with ESRD risk. Statistical analysis was performed using SPSS for Windows version 20.0 (SPSS, Chicago, IL, USA). Two-tailed *P* values of <0.05 were considered significant.

### 2.5. Gene-Gene Interaction Analysis

Gene-gene interactions among the loci were evaluated using multiple dimensionality reduction (MDR) and MDR-permutation testing software (version 1.0 beta). MDR reduces the dimensionality of multilocus information that has reasonable power to identify interactions among two or more loci in relatively small samples and improves the identification of polymorphism combinations associated with disease risk. Average prediction errors were calculated using permutation tests considered significant at *P* < 0.05. Stepwise logistic regression based on backward selection was used to confirm the results of interaction analyses.

## 3. Results

### 3.1. Study Population Demographic and Clinical Characteristics

Table 1 shows the demographic and clinical characteristics of this study population. No significant differences in gender, age, drinking status, and diastolic blood pressure were observed between the two groups, whereas significant differences were observed in other variables (*P* < 0.05).

### 3.2. Distributions of RAAS Gene Polymorphisms and Their Association with ESRD

AGT (M235T and T174M), AGTR1 (A1166C and C573T), ACE (I/D and G2350A), and CYP11B2 (C-344T) genotype distributions were all compatible with Hardy-Weinberg equilibrium for the controls (*P* > 0.05).  Table 2 shows the genotype and allele frequencies of seven SNPs in the two groups. The genotype or allele frequencies for the AGT T174M and AGTR1 (A1166C and C573T) polymorphisms were not significantly different between groups. In addition, those SNPs in dominant and recessive modes were not significantly different (data not shown).

There was a significant association between the AGT M235T polymorphism and ESRD risk, with a mutation carrier having a lower risk (adjusted OR, 0.24; 95% CI, 0.09–0.65; *P* = 0.005). The genotype and allele distributions of ACE I/D and G2350A were significantly different between groups.

For ACE I/D, when genotype II was used as a reference, ID and DD genotypes were apparently associated with a higher ESRD risk (adjusted OR, 1.42; 95% CI, 1.09–1.84; *P* = 0.009; adjusted OR, 1.61; 95% CI, 1.08–2.40; *P* = 0.019, resp.). For ACE G2350A, when the GG genotype was used as a reference, GA and AA genotypes appeared to be associated with a higher ESRD risk (adjusted OR, 1.35; 95% CI, 1.03–1.67; *P* = 0.029; adjusted OR, 1.62; 95% CI, 1.14–2.31; *P* = 0.008, resp.).

Significant associations were found in the CYP11B2 C-344T polymorphism between groups. For CYP11B2 C-344T, the TC genotype compared with the TT genotype was a protective factor for ESRD (adjusted OR, 0.70; 95% CI, 0.54–0.91; *P* = 0.007).

### 3.3. RAAS Gene Polymorphisms Associated with Risk of Different ESRD Causes

We analyzed possible associations between RAAS gene polymorphisms and underlying ESRD etiology. After stratifying the ESRD patients according to different underlying causes for renal disease, an association was indicated for the AGT M235T genotype and glomerulonephritis (adjusted OR, 0.51; 95% CI, 0.31–0.85) but not for diabetic nephropathy, hypertensive nephropathy, systemic nephropathy, or nephropathy for unknown reasons.

There were significant differences in the genotypes of ACE I/D (ID/II, adjusted OR, 1.89; 95% CI, 1.31–2.71) and G2350A (GA/GG, adjusted OR, 1.65; 95% CI, 1.14–2.40; AA/GG, and adjusted OR, 2.04; 95% CI, 1.28–3.28) between patients with diabetic nephropathy and controls. The genotypes of ACE I/D (DD/II, adjusted OR, 2.21; 95% CI, 1.01–4.83) and G2350A (GA/GG, adjusted OR, 1.93; 95% CI, 1.02–3.63; AA/GG, and adjusted OR, 2.41; 95% CI, 1.11–5.22) were also significantly different for patients with hypertensive nephropathy compared with controls. The CYP11B2 genotype was significantly different between patients with systemic nephropathy and controls (adjusted OR, 0.50; 95% CI, 0.25–0.99). No associations were found between patients with different underlying causes of their renal disease and AGT T174M (Table 3).

### 3.4. Evaluations of Gene-Gene Interactions: MDR and LRM

**Figure 1:**
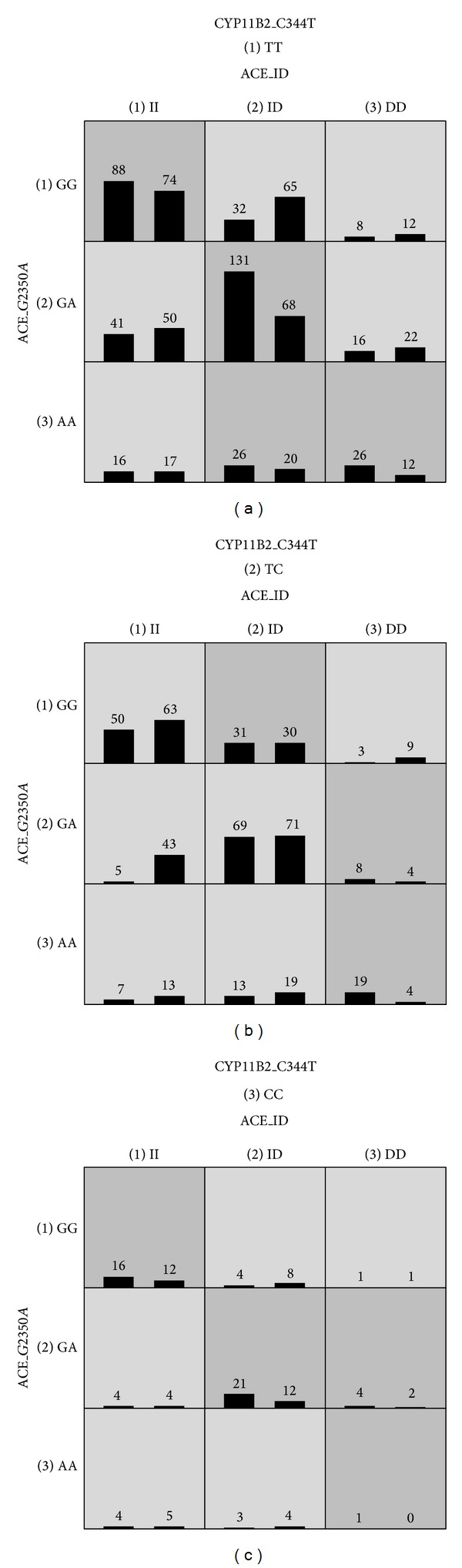
The distribution of cases (left bars) and controls (right bars) for each genotype combination from the three SNPs (ACE I/D, ACE G2350A, and CYP11B2 C-344T) identified in the overall best model by MDR analysis.

**Figure 2:**
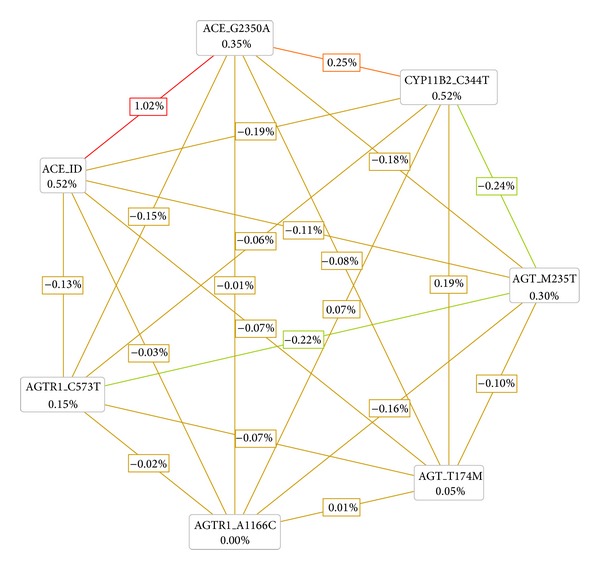
Interaction map for ESRD risk. The values inside boxes indicate information gain (IG) of individual attribute or main effects, whereas values between nodes exemplify IG of pairwise combinations of attributes or interaction effects. A positive entropy (plotted in red or orange) indicates interaction while a negative (plotted in green) indicates redundancy.

Table 4 summarizes the results of an exhaustive MDR analysis for evaluating all possible combinations of the studied polymorphisms. The best overall MDR model included the ACE I/D, ACE G2350A, and CYP11B2 C-344T polymorphisms. This model had a maximum testing accuracy of 56.08% and a maximum cross-validation consistency of 9 out of 10. This model was significant at the 0.01 level, which indicated that a model this good or better would be observed only once in 1,000 permutations; therefore, it was unlikely under the null hypothesis of no association. The distributions for cases and controls for each of the three-locus genotype combinations in the best MDR model are shown in Figure 1.  Figure 2 shows the interaction maps of all genes based on entropy measures among individual variables. A strong interaction effect was found among ACE I/D-ACE G2350A and ACE G2350A-CYP11B2 C-344T, which had information gain values of 1.02% and 0.65%, respectively. Significance in two-way interactions (ACE I/D × ACE G2350A, ACE I/D × CYP11B2 C-344T, and ACE G2350A × CYP11B2 C-344T) was found using a LRM (data not shown). After adjusting for age, gender, body mass index, and smoking status, the interaction of ACE I/D and ACE G2350A SNPs with ESRD was maintained. When the wild-type ACE II-ACE GG genotype was used as a reference, the variant ACE I/D DD-ACE G2350A AA genotype had the greatest ESRD risk (adjusted OR, 3.13; 95% CI, 1.60–6.13; *P* = 0.001; Table 5).

## 4. Discussion and Conclusions

We presented statistical evidence for significant interactions among the ACE I/D, ACE G2350A, and CYP11B2 C-344T genes and the ESRD risk. These results were corroborated by permutation testing. Four genetic polymorphisms in AGT, ACE, and CYP11B2 were found to be significantly associated with the ESRD risk; however, these single SNP analyses did not remain significant after correction for multiple testing (data not shown). Our data also indicated that underlying ESRD etiology differed based on RAAS genes.

Several studies investigated relationships between RAAS gene polymorphisms and ESRD. Many of these focused on the associations of single polymorphisms among RAAS genes with ESRD. The AGT M235T gene polymorphism, which is correlated with circulating and cellular AGT concentrations, has been implicated in ESRD etiology and investigated in epidemiologic studies [18, 19]. The AGTR1 gene was independently associated with renal disease progression and cardiovascular disease [20, 21]. Several studies reported that ACE serum and plasma levels were influenced by ACE I/D [22] and G2350A [23] polymorphisms. An increased ACE level is associated with renal disease pathogenesis. The CYP11B2 C-344T polymorphism was associated with serum aldosterone levels, urinary aldosterone excretion, blood pressure, and left ventricular size and mass [24]. These genetic polymorphisms of key components of the RAAS provide a basis for studying the relationship between genetic variants and the development of renal damage in individual subjects. However, these findings remain controversial, and contributions of interactions on ESRD may provide an explanation. The magnitude of an effect is likely missed if genes are individually examined without considering possible interactions. Evaluating interactions increases the power to detect these effects and also aids in understanding genetic influences on the biological and biochemical pathways that underlie the disease. One study reported on the interactions at the cellular level [25]. This suggests that a deeper insight derived at the statistical level is relevant at the biological level.

An increasing number of studies have focused on interactions, which can be partly attributed due to new statistical theories [26–28]. MDR is a powerful method for analyzing interactions and has been successfully applied in many genetic studies of complex diseases [29, 30]. MDR pools genotypes into “high-risk” and “low-risk” groups to reduce multidimensional data into one-dimension. Our results suggested a three-way interaction between ACE I/D, ACE G2350A, and CYP11B2 C-344T. However, this interaction for susceptibility was not confirmed by LRM, which only found a two-way interaction. A possible reason for this is that MDR did not detect the interaction defined by “deviation from the multiplicative” as in the LRM. Significant results from MDR only implied that the combination of markers contributed to an increased or decreased risk of disease and the effect between these markers could be either multiplicative or a deviation from multiplicative. Additional studies will be needed to establish the underlying mechanisms that explain the possible interactions among ACE I/D, G2350A, and CYP11B2 C-344T polymorphisms for ESRD predisposition.

Recently, many research articles have focused on RAAS gene polymorphisms associated with risk of different ESRD causes. Reis et al. showed that the AGT M235T TT genotype may be linked to a DN risk in the Turkish population [31]. ACE I/D gene polymorphism was associated with DN and reported for Northeast Asians [32]. Song et al. found that CYP11B2 C-344T polymorphism was associated with renal dysfunction progression only in female patients with IgA nephropathy [33]. The C-344T polymorphism was a risk factor for accelerated progression in a Polish population with primary chronic glomerulonephritis [34]. In the present study, we also analyzed possible associations between RAAS gene polymorphisms and underlying ESRD etiology. ACE (I/D and G2350A) was associated with diabetic nephropathy (DN) and hypertensive nephropathy. AGT M235T and CYP11B2 C-344T were associated with glomerulonephritis and systemic nephropathy, respectively. However, the cause of ESRD and interindividual differences in susceptibility remains elusive. More studies are needed to clarify the cause and inter-individual differences in ESRD susceptibility.

Our results suggest that AGT, ACE, and CYP11B2 gene polymorphisms are associated with ESRD and that an interaction effect of ACE I/D, ACE G2350A, and CYP11B2 C-344T polymorphisms may play a more important role than individual factors for ESRD development. A higher ESRD risk was found for the simultaneous occurrence of ACE DD-ACE AA. This investigation was done with Han Chinese patients. The applicability of our results to other ethnic groups is uncertain and warrants further study.

## Figures and Tables

**Table 1:** Characteristics of study subjects.

	Case (*n* = 647)	Control (*n* = 644)	*P* value
Male (%)	301 (46.5%)	266 (41.3%)	0.059
Age (years)	64.4 ± 14.7	65.7 ± 13.6	0.100
Body mass index (kg/m^2^)	22.5 ± 3.9	24.7 ± 3.9	<0.001
Current or former smoker	120 (18.5%)	72 (11.2%)	<0.001
Current or former drinker	68 (10.5%)	60 (9.3%)	0.065
Systolic blood pressure (mmHg)	141.2 ± 34.8	126.4 ± 16	<0.001
Diastolic blood pressure (mmHg)	75.3 ± 11.2	75.9 ± 11.1	0.322
Fasting plasma glucose (mg/dL)	153.6 ± 72.1	98.4 ± 23.5	<0.001
eGFR (mL/min/1.73 m^2^)	5.7 ± 2.6	87.8 ± 14.8	<0.001
BUN (mg/dL)	66.8 ± 19.3	15.6 ± 5.6	<0.001
Uric acid (mg/dL)	7.2 ± 1.5	5.7 ± 1.3	<0.001
Serum creatinine (mg/dL)	9.6 ± 2.6	0.8 ± 0.2	<0.001
Serum total cholesterol (mg/dL)	165.3 ± 35.9	184.7 ± 34.4	<0.001
Serum triglyceride (mg/dL)	157.4 ± 110.7	121.5 ± 98.4	<0.001

**Table 2:** Genotype distribution of the RAAS polymorphisms among ESRD patients and control.

Genotypes	Case	Control	Crude OR (95% CI)	*P* value	Adjusted OR (95% CI)	*P* value
AGT M235T						
CC	466	438	1		1	
CT	169	182	0.87 (0.68–1.12)	0.280	0.78 (0.59–1.03)	0.075
TT	12	24	0.47 (0.23–0.95)	0.036	0.24 (0.09–0.65)	0.005
T allele	193	230	0.81 (0.65–0.99)	0.044	0.70 (0.55–0.89)	0.003
AGT T174M						
CC	508	519	1		1	
CT	134	120	1.14 (0.87–1.5)	0.348	1.11 (0.82–1.51)	0.484
TT	5	5	1.02 (0.29–3.55)	0.973	1.19 (0.33–4.25)	0.786
T allele	144	130	1.12 (0.87–1.43)	0.393	1.11 (0.84–1.46)	0.467
AGTR1 A1166C						
AA	591	589	1		1	
AC	56	55	1.01 (0.69–1.5)	0.941	1.07 (0.7–1.63)	0.761
C allele	56	55	1.01 (0.69–1.48)	0.943	1.06 (0.7–1.61)	0.766
AGTR1 C573T						
CC	336	350	1		1	
CT	267	240	1.16 (0.92–1.46)	0.209	1.23 (0.96–1.59)	0.105
TT	44	54	0.85 (0.55–1.3)	0.450	0.84 (0.52–1.35)	0.478
T allele	355	348	1.02 (0.86–1.21)	0.812	1.05 (0.87–1.27)	0.633
ACE I/D						
II	231	281	1		1	
ID	330	297	1.35 (1.07–1.71)	0.012	1.42 (1.09–1.84)	0.009
DD	86	66	1.59 (1.1–2.28)	0.013	1.61 (1.08–2.4)	0.019
D allele	502	429	1.27 (1.08–1.49)	0.004	1.29 (1.08–1.54)	0.005
ACE G2350A						
GG	233	274	1		1	
GA	299	276	1.27 (1.00–1.62)	0.047	1.35 (1.03–1.76)	0.029
AA	115	94	1.44 (1.04–1.99)	0.028	1.62 (1.14–2.31)	0.008
A allele	529	464	1.23 (1.05–1.44)	0.011	1.31 (1.1–1.56)	0.003
CYP11B2 C-344T						
TT	384	340	1		1	
TC	205	256	0.71 (0.55–0.92)	0.010	0.70 (0.54–0.91)	0.007
CC	58	48	1.07 (0.69–1.67)	0.761	1.07 (0.69–1.68)	0.756
C allele	321	352	0.88 (0.74–1.05)	0.144	0.87 (0.72–1.06)	0.178

Data were expressed as *n* (%) and have been adjusted by gender, age, BMI, and smoking status.

**Table 3:** AGT, AGTR1, ACE, and CYP11B2 gene polymorphisms with risk of different cause of ESRD.

	Diabetic nephropathy (*n* = 256) adjusted^#^ OR (95% CI)	Hypertensive nephropathy (*n* = 84) adjusted^#^ OR (95% CI)	Glomerulonephritis (*n* = 174) adjusted^#^ OR (95% CI)	Systemic nephropathy (*n* = 66) adjusted^#^ OR (95% CI)	Other^&^ (*n* = 67) adjusted^#^ OR (95% CI)
AGT					
M235T					
CT/CC	0.75 (0.52–1.09)	1.07 (0.58–1.98)	0.51 (0.31–0.85)*	0.97 (0.5–1.89)	1.08 (0.59–1.99)
TT/CC	0.33 (0.09–1.14)	—	0.18 (0.02–1.41)	—	0.46 (0.06–3.55)
T174M					
CT/CC	1.26 (0.84–1.88)	1.10 (0.54–2.24)	1.07 (0.66–1.75)	0.79 (0.35–1.77)	1.17 (0.58–2.36)
TT/CC	0.94 (0.17–5.07)	—	0.94 (0.10–8.55)	2.58 (0.29–23.17)	2.13 (0.23–19.54)
AGTR1					
A1166C					
AC/AA	0.79 (0.42–1.47)	0.96 (0.37–2.51)	1.49 (0.81–2.74)	0.72 (0.22–2.43)	1.59 (0.68–3.74)
CC/AA	—	—	—	—	—
C573T					
CT/CC	1.30 (0.92–1.83)	0.96 (0.54–1.70)	1.32 (0.87–1.98)	1.31 (0.69–2.49)	1.00 (0.54–1.84)
TT/CC	0.68 (0.33–1.39)	0.76 (0.26–2.25)	0.59 (0.24–1.46)	1.62 (0.61–4.35)	1.4 (0.55–3.58)
ACE					
ID					
ID/II	1.89 (1.31–2.71)*	1.16 (0.64–2.11)	1.35 (0.89–2.05)	0.83 (0.42–1.61)	1.22 (0.66–2.26)
DD/II	1.71 (0.97–3.00)	2.21 (1.01–4.83)*	1.03 (0.5–2.13)	2.07 (0.93–4.64)	1.99 (0.85–4.65)
G2350A					
GA/GG	1.65 (1.14–2.40)*	1.93 (1.02–3.63)*	1.02 (0.66–1.56)	1.92 (0.97–3.77)	0.91 (0.49–1.68)
AA/GG	2.04 (1.28–3.28)*	2.41 (1.11–5.22)*	1.13 (0.63–2.04)	2.17 (0.9–5.25)	1.12 (0.5–2.53)
CYP11B2					
C-344T					
TC/TT	0.75 (0.52–1.07)	0.58 (0.32–1.04)	0.73 (0.48–1.12)	0.50 (0.25–0.99)*	0.82 (0.44–1.55)
CC/TT	1.11 (0.61–2.00)	0.53 (0.16–1.78)	0.75 (0.34–1.68)	1.55 (0.62–3.85)	2.26 (0.99–5.15)

*P < 0.05, ^#^adjusted for gender, age, BMI, and smoking status; ^&^Others: for example, kidney stone, polycystic kidney disease, and so forth.

**Table 4:** Best gene-gene interaction models identified by the MDR method.

Locus no.	Best model	Testing Bal. Acc. (%)	CVC^†^	*P*-value*
1	ACE I/D	0.5179	6/10	0.6520
2	ACE I/D, ACE G2350A	0.5537	10/10	0.0280
3	ACE I/D, ACE G2350A, CYP11B2 C-344T	0.5608	9/10	0.0060
4	AGTR1 C573T, ACE I/D, ACE G2350A, CYP11B2 C-344T	0.5499	7/10	0.0560
5	AGT M235T, AGTR1 C573T, ACE I/D, ACE G2350A, CYP11B2 C-344T	0.5568	7/10	0.0150
6	AGT M235T, AGT T174M, AGTR1 C573T, ACE I/D, ACE G2350A, CYP11B2 C-344T	0.5290	6/10	0.4100
7	AGT M235T, AGT T174M, AGTR1 A1166C, AGTR1 C573T, ACE I/D, ACE G2350A, CYP11B2 C-344T	0.5227	10/10	0.5500

*Interactions were validated based on 1000 permutations; ^†^CVC: cross-validation consistency.

**Table 5:** Joint effect of ACE I/D and ACE G2350A from ESRD.

Genotypes	Case	Control	Crude OR (95% CI)	*P* value	Adjusted^#^ OR (95% CI)	*P* value^#^
I/D						
II						
G2350A						
GG	154	149	1		1	
GA	67	103	0.63 (0.43–0.92)	0.017	0.68 (0.46–1.05)	0.084
AA	12	22	0.53 (0.25–1.11)	0.090	0.40 (0.16–1.00)	0.050
ID						
G2350A						
GG	50	97	0.5 (0.33–0.75)	0.001	0.52 (0.33–0.84)	0.007
GA	221	151	1.42 (1.04–1.92)	0.026	1.56 (1.09–2.22)	0.015
AA	28	28	0.97 (0.55–1.71)	0.910	1.13 (0.58–2.21)	0.715
DD						
G2350A						
GG	27	35	0.75 (0.43–1.29)	0.297	0.82 (0.43–1.54)	0.536
GA	42	43	0.95 (0.58–1.53)	0.818	1.11 (0.64–1.91)	0.717
AA	46	16	2.78 (1.51–5.13)	0.001	3.13 (1.60–6.13)	0.001

^#^Adjusted for gender, age, BMI, and smoking status.

## Abbreviations

ACE: Angiotensin-converting enzyme
AGT: Angiotensinogen
AGTR1: Angiotensin II type I receptor
CYP11B2: Aldosterone synthase
eGFR: Estimated glomerular filtration rate
ESRD: End-stage renal stage
LRM: Logistic regression model
MDR: Multifactor dimensionality reduction
PCR: Polymerase chain reaction
RAAS: Renin-angiotensin-aldosterone system
RFLP: Restriction fragment length polymorphism
SD: Standard deviation
SNPs: Single nucleotide polymorphisms.
